# Decoding the genomic landscape of *Mammaliicoccus* sp. RAM2 isolated from flacherie-infected *Bombyx mori* L.

**DOI:** 10.1128/mra.01322-24

**Published:** 2025-03-13

**Authors:** Rittick Mondal, Dipanjan Das, Sujan Paul, Atanu Manna, Trishanjan Biswas, Biraj Sarkar, Shubhajit Shaw, Arka Pratim Chakraborty, Amit Kumar Mandal

**Affiliations:** 1 Chemical Biology Laboratory, Department of Sericulture, Raiganj University 561306, Raiganj, West Bengal, India; 2 Mahatma Gandhi Medical Advanced Research Institute (MGMARI), Sri Balaji Vidyapeeth (Deemed to be University), Pondicherry, India; 3 Faculty of Allied Health Sciences (FAHS), The ICFAI University, Kamalghat, Tripura, India; 4 Department of Botany, Raiganj University 30163, Raiganj, West Bengal, India; Wellesley College Department of Biological Sciences, Wellesley, Massachusetts, USA

**Keywords:** genomics

## Abstract

Species of the genus *Mammaliicoccus* are recognized as opportunistic zoonotic pathogens. Here, we report the draft genome sequence of *Mammaliicoccus* sp. RAM2, isolated from a flacherie-infected *Bombyx mori* L. (Nistari race) from Raiganj University, India (25.6071° N, 88.1306° E), which offers valuable genetic insights for precise species-level identification.

## ANNOUNCEMENT

The genus *Mammaliicoccus* comprises non-motile, gram-positive bacteria that are widely distributed in human samples, agricultural settings, and wildlife habitats. It contributes to antibiotic resistance gene transmission and has been shown to infect silkworms, posing public health risks (1, 2).

*Mammaliicoccus* sp. strain RAM2 was isolated from the *B. mori* L larval hemolymph. The collected hemolymph was serially diluted in PBS, spread on mannitol-salt agar (MSA) plate (composition: proteose peptone, 10.0 g; sodium chloride, 75.0 g; D-mannitol, 10.0 g; HM peptone B, 1.0 g, phenol red, 0.025 g, agar, 15.0 g, distilled water, 1 L, pH 7.4) (3) and incubated overnight at 30°C. After incubation, single colonies were picked and transferred to fresh MSA plates to obtain pure cultures. These cultures were maintained on MSA plates for up to 12 generations before being preserved at −80°C in 20% glycerol.

The genomic DNA of RAM2 was extracted by the standard phenol-chloroform method (4). The paired-end libraries were constructed prior to sequencing using the Illumina NovaSeq 6000 platform (Neuberg Diagnostics Pvt. Ltd., Ahmedabad, India). The DNA library was prepared using the KAPA HyperPlus Kit (Roche #07962428001). The final DNA libraries were quantified using the Qubit 4.0 fluorometer (ThermoFisher #Q33238) using the DNA HS assay kit (ThermoFisher #Q32851) following the manufacturer’s protocol. The insert size of the library was identified using the TapeStation 4150 system (Agilent) utilizing highly sensitive D1000 Screentapes (Agilent # 5067–5582) following the manufacturer’s protocol, yielding 23,856,714 reads with a 2 × 150 bp paired-end read length.

**Fig 1:**
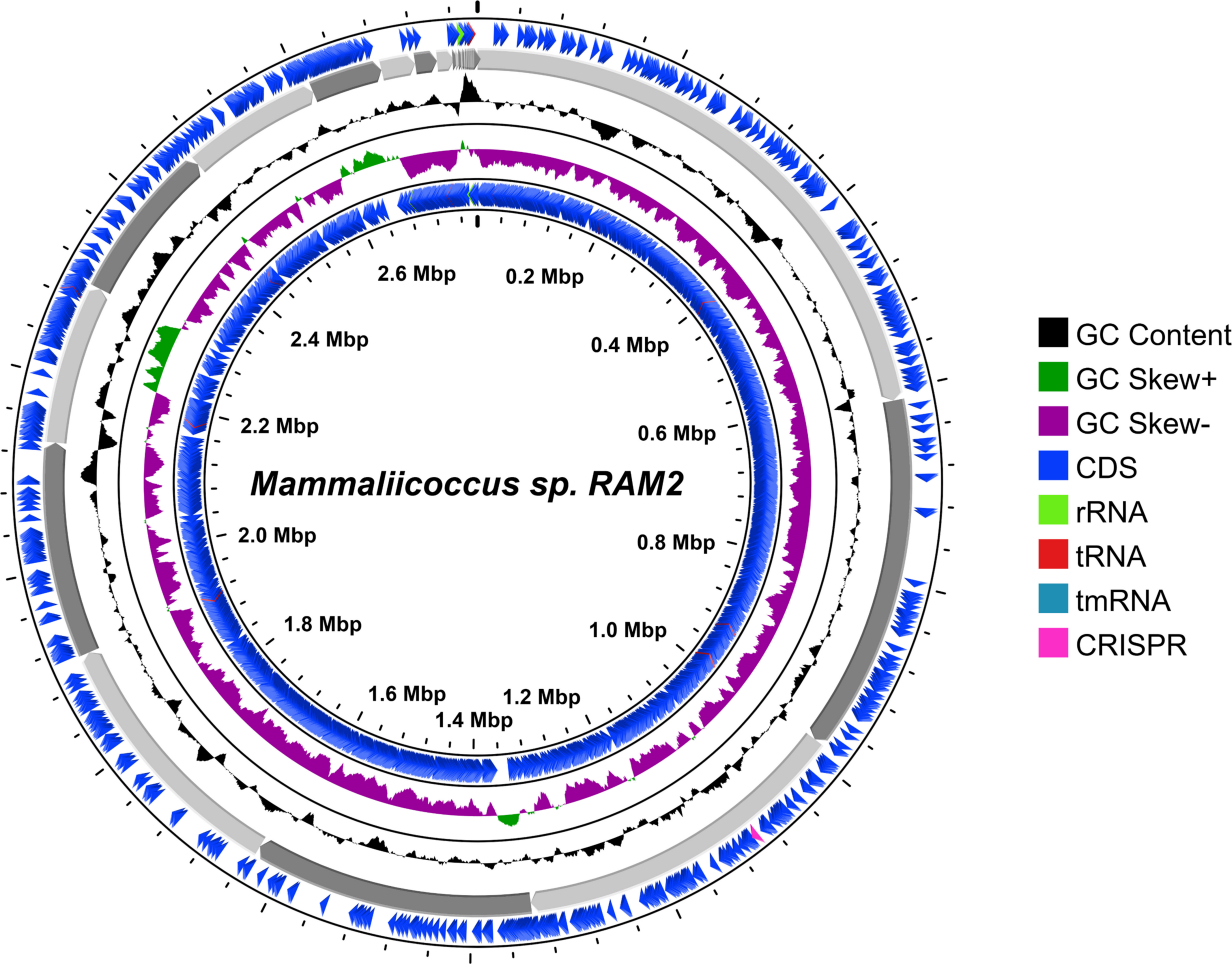
The genome map *Mammaliicoccus* sp. strain RAM2, generated using the CGView Server. The blue arrows represent CDSs, gray arrows show contigs, the black plot indicates GC content, and the green and magenta plot represents CG skew + and −, respectively.

Quality assessment of the raw fastq reads of the sample was performed using FastQC v.0.11.9 (default parameters) (5). The raw fastq reads were preprocessed using Fastp v.0.23.4 (6) (parameters: *--length_required 50—correction --trim_poly_g --qualified_quality_phred 30—unqualified_percent_limit 30—average_qual 30*). Processed data were re-assessed using FastQC. The processed reads were *de novo-*assembled using Unicycler v.0.4.4 (7) with default parameters. Estimations of the completeness and rate of contamination of the assembled genome were done using CheckM2 v.1.0.1 (8). The annotation was carried out via the NCBI Prokaryotic Genome Annotation Pipeline (PGAP) v6.9 with the methods best-placed reference protein set and GeneMarkS-2+ (9). The assembly produced a draft genome sequence encompassing 42 contigs. The *N*_50_ value is 294,727 bp, and the *L*_50_ count is 4. The estimated genome size is 2,786,715 bp, with a G + C content of 32.39% and 1107.55 x coverage. A total of 2,765 coding sequences were annotated, including three rRNA genes (one 5S, one 16S, and one 23S rRNA genes) and 50 tRNA genes. Finally, a genome map of RAM2 was generated using CGView Server (https://cgview.ca/) (10) (Fig. 1). The presence of antibiotic resistance genes such as *salC*, *sdrM*, *sepA*, *vanY* (in the *vanF* cluster), and *vanT* (in the *vanG* cluster) within the genome provides insights into the isolate’s genotypic diversity, host–microbe interactions, molecular pathogenesis, and the emerging threat of antimicrobial resistance (AMR). This underscores the need for continued surveillance and the development of novel therapies to combat AMR.

## Data Availability

This whole-genome shotgun project data have been deposited at NCBI under the accession number JBJNWI000000000. The version described in this paper is the first version, JBJNWI010000000. The BioSample and BioProject accession numbers are SAMN45041942 and PRJNA1190400, respectively. The raw data are available from the Sequence Read Archive (SRA) under the accession number SRR31595130. The isolated Mammaliicoccus sp. strain RAM2 was deposited at the MACS Collection of Microorganisms (MCM), Agharkar Research Institute, India, under the accession number "MCM-B-1535."
